# A New Method of Reducing Dislocation of the Jaw

**Published:** 1895-04

**Authors:** Eugene S. Talbot


### Domestic Correspondence.

### A NEW METHOD OF REDUCING DISLOCATION OF THE JAW.

To the Editor:

Sir,—Under the head of "Selections" in the February number of the International Dental Journal appears the following:

"Dr. Roth seats the patient in an ordinary chair, and stands before him with one foot placed slightly to the right side and the other just in front of the patient and in the middle line. He then flexes himself at the hips and causes the patient to lean forward and to place his forehead at the middle of the operator's sternum,—but this position varies with the size of the patient's head. The operator now flexes his neck so that his chin grips the patient's head about the upper part of the occipital bone, thus acquiring a firm hold with the head well under control between his chin and chest. Now the thumbs, protected in the usual manner, are placed in the patient's mouth, and the fingers of both hands grasp the lower jaw.—*The Medical Age.*"

The late Professor Wildman, of the Pennsylvania College of Dental Surgery, taught this method in 1870–71. The difference being that a common low chair was used so that the operator standing in front and above can obtain a good leverage with his arms and hands. The patient leaned forward and the operator over his patient readily carried the jaw back into place. I have practised this method ever since. The only difference between the method of the late Professor Wildman and the "new method" seems to be in the operator holding the head of the patient with his chin. In connection with the description of reducing a dislocation of the lower jaw he used to tell the following story: A lady living near a prominent surgeon in Philadelphia was a chronic scold; when the mania came on she would continue her scolding until her lower jaw slipped forward and out of the sockets. She would visit her surgeon who would replace it without difficulty. Finally her scolding spells became so frequent and the necessity of a surgical operation so often that the surgeon became annoyed. Seeing her enter his office one day with her mouth open and jaw protruding, he seized a chair, invited her to be seated, and as she did so the chair was pulled from under her, and the jar she received upon striking the floor threw the jaw into its natural position. The surgeon was never troubled afterwards.

Respectfully,

EUGENE S. TALBOT.

